# Variations in Hormones and Antioxidant Status in Relation to Flowering in Early, Mid, and Late Varieties of Date Palm (*Phoenix dactylifera*) of United Arab Emirates

**DOI:** 10.1155/2015/846104

**Published:** 2015-06-16

**Authors:** Abdul J. Cheruth, Shyam S. Kurup, Sreeramanan Subramaniam

**Affiliations:** ^1^Department of Aridland Agriculture, College of Food and Agriculture, P.O. Box 15551, Al-Ain, UAE; ^2^School of Biological Sciences, Universiti Sains Malaysia (USM), Minden Heights, 11800 Georgetown, Penang, Malaysia

## Abstract

The present study was carried out to assess the status of various hormones responsible for the flower induction of Nagal, Lulu, and Khalas date palm varieties in UAE. The nonenzymatic antioxidant compounds and the antioxidant enzymatic activities at preflowering, flowering, and postflowering stages of the date palm varieties were quantified. The ABA and zeatin concentrations were found to be significantly higher during the preflowering stage but gradually decreased during the flowering period and then increased after the flowering stage. Gibberellic acid (GA) concentrations were significantly higher in the early flowering varieties and higher levels of ABA may contribute to the delayed flowering in mid and late varieties. The results on hormone profiling displayed a significant variation between seasons (preflowering, flowering, and postflowering) and also between the three date palms (early, mid, and late flowering varieties). Ascorbic acid (AA) concentration was low at the preflowering stage in the early flowering Nagal (0.694 mg/g dw), which is similar with the late flowering Lulu variety (0.862 mg/g dw). However, Khalas variety showed significantly higher amount of AA content (7.494 mg/g dw) at the preflowering stage when compared to other varieties. In flowering stage, Nagal (0.814 mg/g dw) and Lulu (0.963 mg/g dw) were similar with respect to the production of AA, while the mid flowering variety showed significantly higher amount of AA (9.358 mg/g dw). The Khalas variety produced the highest tocopherol at 4.78 mg/g dw compared to Nagal and Lulu, at 1.997 and 1.908 mg/g dw, respectively, during the preflowering stage. In Nagal variety, the content of reduced glutathione (GSH) at the preflowering stage was 0.507 mg/g dw, which was not significantly different from the flowering and postflowering stages at 0.4 and 0.45 mg/g dw, respectively. The GSH was significantly higher in Khalas compared to Nagal and Lulu varieties, at 1.321 mg/g dw in the preflowering phase followed by 3.347 mg/g dw and 2.349 mg/g dw at the flowering and postflowering phases, respectively. Catalase activity increased with different stages of growth. The lowest catalase activity was observed at the preflowering stage in Khalas (0.116), with similar observations noted during flowering (0.110) and postflowering stage. This study provides an insight into the possible roles of endogenous hormones and antioxidants and in the activities of antioxidant enzymes in the regulation of flower development in date palm varieties.

## 1. Introduction

Dates have been a staple food of the Middle East and parts of South Asia for thousands of years. Dates are believed to have originated from the Persian Gulf and have been grown since ancient times dating back to prehistoric Egypt [1]. More than 1,000 varieties of dates have been recorded [2]. It is very difficult to discriminate closely related date palm cultivars and clones for genetic diversity studies. Cultivar identification is usually based on morphological parameters of plant and fruits. But due to unreliability, this often creates problems and may not correlate with the genotype of the tree [3].

Date palm cultivation depends on its climatic requirements. The successful cultivation of date palm requires long summer season with high day and night temperatures, a mild winter without frost, the absence of rain during flowering and fruit setting, and low relative humidity with sufficient sunshine [4]. It was estimated that the finest date palm varieties required 3,300 units of heat (based on 10°C) for full maturity of its berries.

The timing of the transition from vegetative growth to flowering is of paramount importance in agriculture and plant breeding, since flowering is the first step of sexual reproduction [5]. The antioxidant metabolism behind plant defense mechanism has been the area of interest for a long time in a variety of crops [6–10], but little attention is given to flowering-related changes in terms of antioxidant status. Significant variations in antioxidant properties were observed in different development stages of* Carthamus tinctorius* flowers, and the highest antioxidant activity was obtained in stage III (full flowering), while phenolic composition reached its maximum at stage II (flower formation) [11]. There are reports on the antioxidant characteristics of date fruit [12] and antioxidant capacity, antioxidant compounds, and antioxidant enzyme activities in date cultivars during development and ripening [13]. Studies have reported that the date palm fruit might be a good source of secondary active components and has a potent ability to suppress free radicals [14–16].

Usually, date palm is cultivated in arid and semiarid regions which are characterized by long and hot summers, low rainfall, and very low relative humidity level during the fruit ripening period. The temperature requirements are important for determining growth, flowering, and fruit maturation. Date palm flowering is initiated after a cold period especially when the temperature becomes high enough and reaches a level known as the flowering zero. The variation in this temperature level depends on the date palm varieties and local climatic conditions. Flowering temperatures represent the average daily temperatures from initiation till the end of the flowering period, while the fruiting period of date palm commences at fruit set and ends at fruit maturation [17]. Flower bud initiation and time of fruit set in date palm are important factors determining the commercial production of date fruits. Accordingly, there are three flowering forms in date palms, based on the early, mid, and late season varieties. Additional characterizations are based on flowering, fruit set, and qualitative criteria. The early seasonal production is important in garnering premium market price. Hence, this study is crucial for determining the basic physiological mechanisms underlying the flowering behaviour in early, mid, and late varieties of date which could form the basis to develop the early-bearing varieties.

The objectives of the present study were to assess the status of hormones responsible for flower induction and to quantify the nonenzymatic antioxidant compounds and antioxidant enzymatic activities at the preflowering, flowering, and postflowering stages of the date palm.

## 2. Materials and Methods

### 2.1. Planting Materials and Sampling

The experimental plants were sampled from the College of Food and Agriculture experimental farm at the UAE University located at Al-Foah, Al-Ain, United Arab Emirates. In each variety, three date palms were identified with three replicates. The varieties of date palms studied were classified as early-season cv. Nagal, mid-season cv. Khalas, and late-season cv. Lulu. These date palm varieties are in the same age group and were in a healthy condition with stabilized production. Normal cultural practices were followed for all the trees according to the recommended package of practice. The leaf sampling for hormonal profiling and analysis of antioxidant metabolism were conducted at the middle whorl in three phases of growth and development, namely, preflowering, flowering, and postflowering.

### 2.2. Hormone Profiling

The hormones gibberellic acid (GA), IAA, auxin, and ABA were determined based on Kelen et al. [18]. Ten (10) g of fresh tissue sample which was homogenized in 70% methanol was stirred overnight at 4°C. The extract was filtered through Whatman filter paper (No. 1) and evaporated under vacuum. The pH of the aqueous phase was adjusted to 8.5 using 0.1 M phosphate buffer. Later the aqueous phase was partitioned using ethyl acetate twice. The ethyl acetate phase was removed and the aqueous phase pH was adjusted to 2.5 using 1 N hydrochloric acid (HCI). The hormones were partitioned into diethyl ether three times. The diethyl ether phase comprising the hormones was vacuum-dried. The residue obtained was dissolved in 1 mL of methanol and stored at 4°C for further analysis. The extracted phytohormones were separated by HPLC (Shimadzu) on a reverse phase C18 column (250 × 4.60 mm, 5 micron) in an isocratic elution mode using a mobile phase consisting of acetonitrile: water (26 : 74) with 30 mM phosphoric acid [18]. The pH was maintained at 4 using 1 N sodium hydroxide. A temperature of 25°C was maintained for the column. The flow rate was 0.8 mL/min and the elution of the phytohormones was monitored at 208, 265, 270, and 280 nm.

### 2.3. Antioxidant Compounds

#### 2.3.1. Ascorbic Acid

Ascorbic acid content was assayed as described by Omaye et al. [19]. One (1) gram of fresh material was ground in a pestle and mortar with 5 mL of 10 percent TCA, and the extract was centrifuged at 3500 rpm for 20 minutes. The pellet was extracted twice with 10% TCA and the supernatant topped up to 10 mL and used for the estimation. One (1) mL of DTC reagent (2,4-dinitrophenylhydrazine-thiourea-copper sulphate reagent) was added to 0.5 mL of the extract and mixed thoroughly. The tubes were incubated at 37°C for 3 hours, followed by the addition of 0.75 mL of ice-cold 65% H_2_SO_4_. The tubes were then allowed to stand at 30°C for 30 minutes. The resulting colour was measured at 520 nm with a spectrophotometer (U-2001-Hitachi). The ascorbic acid content was determined using standard ascorbic acid and expressed in milligrams per gram dry weight.

#### 2.3.2. *α*-Tocopherol

*α*-Tocopherol activity was assayed according to Baker et al. [20]. Five hundred (500) milligrams of fresh tissue was homogenized with 10 mL of a mixture of petroleum ether and ethanol (2 : 1.6 v/v) and the extract was centrifuged at 10,000 rpm for 20 minutes, with the supernatant used for estimation of *α*-tocopherol. Using one (1) mL of extract, 0.2 mL of 2 percent 2,2-dipyridyl in ethanol was added and mixed thoroughly and kept in the dark for 5 minutes. The resulting red colour was diluted with 4 mL of distilled water and mixed well. The resulting colour in the aqueous layer was measured at 520 nm and *α*-tocopherol content was calculated using the standard *α*-tocopherol.

#### 2.3.3. Reduced Glutathione

Reduced glutathione was assayed based on Griffith and Meister [21]. Two hundred (200) milligrams of fresh material was ground in a pestle and mortar with 2 mL of 2% metaphosphoric acid. The extract was centrifuged at 17,000 rpm for 10 minutes. The supernatant was neutralized by adding 0.6 mL of 10% sodium citrate. One (1) mL of the assay mixture was prepared by adding 100 *μ*L extract, 100 *μ*L distilled water, 100 *μ*L DTNB, and 700 *μ*L NADPH. The assay mixture was stabilized at 25°C for 3-4 minutes. Then 10 *μ*L of glutathione reductase (sigma) was added and the absorbance was measured at 412 nm.

### 2.4. Estimation of Antioxidant Enzyme Activities

#### 2.4.1. Ascorbate Peroxidase (APX)

Ascorbate peroxidase was extracted and estimated by the method of Asada and Takahashi [22]. Five hundred (500) milligrams of fresh plant tissue was ground in a pestle and mortar under liquid nitrogen and 10 mL of 50 mM potassium phosphate buffer (pH 7.0) containing 1 mM EDTA, 1% PVP, and 1 mM ascorbic acid. The homogenate was filtered through a double layered cheese cloth and centrifuged at 15,000 rpm for 20 minutes at 4°C. The supernatant was used as source of enzymes. One (1) mL of reaction mixture contained 50 mM potassium phosphate buffer (pH 7.0), 0.5 mM ascorbic acid, 0.1 mM H_2_O_2,_ and 200 *μ*L of enzyme extract. The absorbance value was read as a decrease at 290 nm against the blank, based on the nonenzymatic oxidation of ascorbic acid by H_2_O_2_ (extinction coefficient 2.9 mM^−1^ cm^−1^). The enzyme activity was expressed in units mg^−1^ protein (*U* = change in 0.1 absorbance min^−1^ mg^−1^ protein).

#### 2.4.2. Superoxide Dismutase (SOD)

Crude enzyme extract was prepared for the superoxide dismutase assay based on Beauchamp and Fridovich [23]. One (1) gram of fresh tissue was homogenized with 10 mL of ice-cold 50 mM sodium phosphate buffer (pH 8.0) containing 1 mM PMSF. The extract was filtered through a double layered cheese cloth. The extract was centrifuged at 12,500 rpm for 20 minutes at 4°C. The supernatant was saved and made up to 10 mL with extraction buffer and used for estimation of the SOD enzyme activity. The enzyme samples were determined using the Bradford method [24]. The reaction mixture contained 1.17 × 10^−6^ M riboflavin, 0.1 M methionine, 2 × 10^−5^ potassium cyanide, and 5.6 × 10^−5^ M nitroblue tetrazolium salt (NBT) dissolved in 0.05 M sodium phosphate buffer (pH 7.8). The mixture was illuminated in glass test tubes by two sets of Philips 40 W fluorescent tubes. Illumination initiated the reaction, which was conducted at 30°C for one hour. The absorbance value was measured at 560 nm and superoxide dismutase activity was expressed in units. One (1) unit is defined as the amount of change in the absorbance by 0.1 per hour per milligram protein under the assay condition [25].

#### 2.4.3. Catalase (CAT)

The activity of enzyme catalase was measured based on Chandlee and Scandalios [26] with some modifications. Five hundred (500) milligrams of frozen material was homogenized in 5 mL of ice-cold 50 mM sodium phosphate buffer (pH 7.5) containing 1 mM PMSF. The extract was centrifuged at 4°C for 20 minutes at 12,500 rpm. The supernatant was used for the enzyme assay. The assay mixture contained 2.6 mL of 50 mM potassium phosphate buffer (pH 7.0) 0.4 mL 15 mM H_2_O_2_ and 0.04 mL of the enzyme extract. The decomposition of H_2_O_2_ was followed by the decline in absorbance at 240 nm. The enzyme activity was expressed in units of 1 mM of H_2_O_2_ reduction per minute per mg protein.

#### 2.4.4. Peroxidase (POX)

Peroxidase was assayed using the method of Kumar and Khan [27]. The assay mixture contained 2 mL of 0.1 M sodium phosphate buffer (pH 6.8), 1 mL of 0.01 M pyrogallol, 1 mL of 0.005 M H_2_O_2_, and 0.5 mL of enzyme extract. The solution was incubated for 5 min at 25°C before termination by the addition of 1 mL 2.5 N H_2_SO_4_. The amount of purpurogallin developed was determined by measuring the absorbance at 420 nm against the blank with 2.5 N H_2_SO_4_. The activity was expressed in unit mg^−1^ protein. One (1) unit is defined as the change in the absorbance by 0.1 min^−1^ mg^−1^ protein.

### 2.5. Statistical Analysis

Each treatment was analysed with at least three replicates. The standard error (SE) was calculated and data were expressed as the ±SE of three replicates. Values that are not sharing a common superscript differ significantly at *p* ≤ 0.05 (DMRT).

## 3. Results

### 3.1. Hormone Profiling

The hormones studied were gibberellic acid, indole acetic acid, zeatin, and abscisic acid (Table 1). The ABA concentration showed changes according to the stages of growth as it was high during the preflowering stage but gradually decreased during the flowering period. However, ABA increases again after the flowering stage. IAA concentration was high during flowering induction period in all the date palms. The GA concentration was significantly high in early flowering varieties of the date palm.

### 3.2. Nonenzymatic Antioxidant Contents

Ascorbic acid (AA) concentration was the lowest during preflowering stage in Nagal palm (0.694 mg/g dw), which is on a par with late flowering Lulu variety (0.862 mg/g dw) (Table 2). Lower levels of AA are a prerequisite for the production of flower induction hormones such as IAA and gibberellic acid (GA). However, the mid flowering Khalas variety showed significantly high amount of AA content (7.494 mg/g dw) at the preflowering stage when compared to the other date palm varieties. This could be attributed to the antioxidant potential of AA which is evident due to the severe winter which favours the many-fold increase in the biosynthesis of AA. By the time of flowering, the AA concentration increases as the season changes to winter. AA protects plants against abiotic stress. In the flowering stage, Nagal (0.814 mg/g dw) and Lulu (0.963 mg/g dw) were found to be on a par with the production of AA, while the mid flowering variety showed a significantly high amount of AA (9.358 mg/g dw). There is a transient rise in the AA content which could be seen in flowering and postflowering stages in all the varieties except for Khalas which showed a marginal decrease (9.168 mg/g dw) in the postflowering phase.

The Khalas variety showed the highest content of tocopherol with 4.78 mg/g dw compared to Nagal and Lulu, at 1.997 and 1.908, respectively, at the preflowering stage (Table 2). A significant increase in the content of tocopherol was observed at the flowering stage in all varieties. This increase in tocopherol could be attributed to the low temperature stress observed at the flowering stage in the date palm. Tocopherol is a strong antioxidant that can induce tolerance to stress [28]. Irrespective of the varieties, there were significant increases in tocopherol contents in the varieties, with Khalas showing the highest value (7.122 mg/g dw), followed by Nagal (5.284 mg/g dw) and Lulu (4.199 mg/g dw).

In Nagal variety, the content of reduced glutathione (GSH) at the preflowering stage was 0.507 mg/g dw, which was not significantly different from the flowering and postflowering stages, at 0.4 and 0.45 mg/g dw, respectively (Table 2). In Khalas, the amount of GSH was significantly higher than the Nagal and Lulu varieties, at 1.321 mg/g dw at the preflowering phase followed by 3.347 mg/g dw at flowering and 2.349 mg/g dw at postflowering phases. The Lulu variety displayed similar preflowering contents with that of the Nagal variety, at 5.13 mg/g dw, with both recording 0.419 and 0.619 mg/g dw at the postflowering phase, respectively.

### 3.3. Antioxidant Enzyme Activities

Catalase activity increased with different stages of growth. The lowest value was observed in preflowering Khalas (0.116), which maintained the same status at the flowering (0.110) and postflowering stages (Table 3).

Peroxidase (POD) and ascorbate peroxidase (APX) activities were high at the preflowering stage and declined during the flowering and postflowering stages. A high POD content was observed in Lulu (0.729) followed by early flowering Nagal (0.559) and Khalas (0.165) varieties during preflowering stage. Apart from this superoxide dismutase (SOD) activity displayed a similar trend, with the exception of the mid flowering variety (Table 3).

## 4. Discussion

High levels of ABA may contribute to the delayed flowering in the mid and late varieties. However, the number of flowers per panicle in the ABA treated in litchi plants increased when exogenous ABA was added prior to the emergence of the panicle primordia [29]. Previously, it has been suggested that a low GA level is important for transporting nutrients from the vegetative organs into reproductive organs [6].

Usually, various events take place during flower bud opening and senescence, with changes in concentration of endogenous plant growth regulators and secondary metabolites due to well-defined sequence such as cell division, cellular differentiation, and shifts in membrane permeability, cell elongation, and a wide range of gene expression in association [30, 31]. Little information is available on hormonal changes during different stages of flowering in date palm. Therefore, this study focused on the importance of hormonal profiling and enzymatic activities of three different seasons of date palm varieties. In higher plants, the timing of the transition from the vegetative to the reproductive phase is essential to ensure reproductive success. Flowering is sensitive to physiological parameters and to current environmental conditions, including N availability and the presence of competitors [32]. Flowering time is controlled by external and internal factors that are integrated in a complex gene regulatory network that ensures the expression of flowering genes, resulting in flower formation [33, 34]. Environmental factors that regulate flowering include day length, light, and temperature. The plant hormone GA is an important internal factor that controls flowering mechanisms.

Transition to flowering, inflorescence differentiation, and leaf morphogenesis are quantitative and cumulative processes. Lifschitz et al. [35] reported a systemic mechanism for coordinating growth and termination in flowering plants based on the florigen and antiflorigen model. The time of blossoming and pollination varies according to the different types of date palms. For developing an early bearing variety of date palm, the physiology of flowering should be explored. Therefore, this study attempted to reveal the hormonal and antioxidant metabolism of three distinct varieties of date palm. The results on hormone profiling showed a significant variation between seasons (pre flowering, flowering, and postflowering) and also between the three varieties (early, mid, and late flowering varieties).

Similar trends were observed in the postflowering stages of all the varieties. Ascorbic acid (AA) protects plants against abiotic stress. Previous studies suggested that this antioxidant is also involved in the control of flowering [36]. The antioxidant metabolism of higher plants undergoes alterations according to a seasonal cycle. Minimum thresholds in antioxidant activities could be crucial for scavenging free radicals generated during the winter season. Increased tocopherol content has been correlated in the response of photosynthetic tissues to a variety of abiotic stresses [37]. In the present study, the postflowering stage of the crop overlaps with the summer season leading to high fluctuations in temperature to protect the plants from the oxidative stress.

Flowering is induced not only by photoperiod but also by chilling stress, as observed in the date palm, where a specific period of chilling is an indispensable factor for flower induction. The GSH biosynthesis rate increased due to stress-inducible activation of glutamylcysteine synthetase at the posttranscriptional levels [38], which may have enhanced the stress-associated promotion of flowering. Hence, this finding may benefit the date palm cultivation industry since flowering is induced only during low-temperature months. Chilling stress is known to cause an oxidative stress and to induce changes in the GSH content of plants [39, 40]. This was observed in this study where noticeable changes in the content of GSH occurred in all the three varieties.

Abassi et al. [41] reported a burst of catalase activity during bud development which peaked during fruit set in apple, contrary to the results obtained from the mid flowering variety of the date palm in this study. The results on early and late flowering are in conformity with Abassi et al. [41], as there was a significant increase of CAT activity during early flowering at all the three stages, except in late flowering stage. Hence, it was possible that the increases in catalase activity at these stages could have resulted from the accumulation of H_2_O_2_ due to a higher rate of respiration which in turn might have resulted from the low temperature prevailing during the flowering season [42].

There were no significant differences in POD activity in the early, mid, and late flowering varieties. But the activity declined significantly at the postflowering stage in all three varieties. POD activity changed significantly during apple bud and fruit development [41]. POD activity was higher prior to bud break and then declined during flower bud and fruit development. Wang et al. [43] reported on the POD activity profile during early stages of vegetative bud development. They found high POD activity in dormant buds, which then declined during bud break but increased during bud growth.

Date fruits undergo many physical and compositional changes during maturation. Some of these changes, such as the decrease in the concentration of tannins, ascorbic acid, and b-carotene, directly affect their antioxidant capacity [44]. The concentration of antioxidant compounds is constitutive and variable from species to species and is also variable considering the development of the plant tissue [44]. The variations in the levels of antioxidant compounds are related with floral developmental processes and especially with the last step of senescence. The plant defense mechanisms against external and internal senescence associated factors were extremely complex [45]. Several studies have demonstrated that free radicals are formed in dormant buds, where their removal seems to be associated with bud break. Protection against free oxygen radicals is achieved through the action of antioxidant compounds and enzymes. There were significant changes in antioxidant enzymes (catalase, peroxidase, and ascorbate peroxidase), sulfhydryl compounds, and glutathione content in flower buds of two apricot cultivars with different chilling requirement, during the winter season [46].

Recently there are many reports on the antioxidant activity and their possible role in health related effects of date fruits [47–49], but our study is different from those aspects. Even though we studied the antioxidants, their role in flowering is quite new aspect. This study provides an insight into the possible role of endogenous hormones, antioxidant contents, and activities of antioxidant enzymes in regulating flower development in date palm varieties. Future work will evaluate the importance of these metabolites in all cultivars of the early, mid, and late flowering date palm varieties at various stages of flower development.

## Figures and Tables

**Table 1:** Variation in hormone concentrations at different stages of growth in the early, mid, and late varieties of date palm.

Variety	Growth stage	GA (*μ*g/g)	IAA (*μ*g/g)	Zeatin (*μ*g/g)	ABA (*μ*g/g)
Early flowering (Nagal)	Preflowering	32.57 ± 2.4^a^	1.59 ± 0.15^a^	6.93 ± 0.74^b^	1.30 ± 0.17^a^
	Flowering	30.28 ± 2.5^b^	0.72 ± 0.08^b^	5.12 ± 0.63^b^	0.90 ± 0.09^b^
	Postflowering	31.24 ± 3.5^a^	0.97 ± 0.12^a^	7.24 ± 0.58^a^	0.89 ± 0.16^b^

Mid flowering (Khalas)	Preflowering	19.60 ± 1.9^c^	1.77 ± 0.15^a^	5.64 ± 0.52^a^	1.97 ± 0.21^a^
	Flowering	24.58 ± 2.2^b^	1.01 ± 0.16^b^	4.52 ± 0.84^b^	1.25 ± 0.15^b^
	Postflowering	28.52 ± 2.6^a^	1.82 ± 0.17^a^	4.85 ± 0.65^a^	1.45 ± 0.22^b^

Late flowering (Lulu)	Preflowering	15.33 ± 1.9^c^	0.05 ± 0.02^c^	2.79 ± 0.43^a^	0.63 ± 0.11^a^
	Flowering	21.06 ± 2.0^b^	0.13 ± 0.03^b^	1.88 ± 0.38^b^	0.92 ± 0.17^a^
	Postflowering	23.12 ± 2.3^a^	0.21 ± 0.02^a^	2.30 ± 0.41^a^	0.62 ± 0.14^a^

GA: gibberellic acid, IAA: indole acetic acid, and ABA: abscisic acid.

Values are means of 3 replications in each variety. Means with different letters in the same variety indicate significantly different values at *p* < 0.05.

**Table 2:** Variation in nonenzymatic antioxidant contents at different stages of growth in the early, mid, and late varieties of date palm.

Variety	Growth stage	Ascorbic acid Mg/g DW	*α*-Tocopherol	GSH
Nagal	Preflowering	0.694 ± 0.072^b^	1.997 ± 0.430^c^	0.507 ± 0.056^a^
	Flowering	0.814 ± 0.125^a^	4.369 ± 0.539^b^	0.400 ± 0.274^a^
	Postflowering	1.930 ± 0.185^b^	5.284 ± 1.102^a^	0.450 ± 0.035^b^

Khalas	Preflowering	7.494 ± 0.491^b^	4.788 ± 0.513^b^	1.321 ± 0.074^c^
	Flowering	9.358 ± 0.610^a^	6.913 ± 0.434^b^	3.347 ± 0.054^a^
	Postflowering	9.168 ± 0.880^a^	7.122 ± 0.690^b^	2.349 ± 0.079^b^

Lulu	Preflowering	0.862 ± 0.123^b^	1.908 ± 0.342^b^	0.513 ± 0.045^a^
	Flowering	0.963 ± 0.101^b^	2.392 ± 0.355^b^	0.491 ± 0.021^a^
	Postflowering	1.532 ± 0.049^a^	4.199 ± 0.836^a^	0.619 ± 0.030^b^

GSH: reduced glutathione.

Means with different letters in the same variety indicate significantly different values at *p* < 0.05.

**Table 3:** Variation in antioxidant enzyme activities at different stages of growth in the early, mid, and late varieties of date palm.

Variety	Growth stage	SOD	CAT	POX	APX
Nagal	Preflowering	0.433 ± 0.067^a^	0.665 ± 0.088^c^	0.559 ± 0.042^a^	0.830 ± 0.108^a^
	Flowering	0.353 ± 0.045^b^	1.338 ± 0.044^b^	0.483 ± 0.025^a^	0.467 ± 0.041^b^
	Postflowering	0.384 ± 0.017^b^	1.755 ± 0.187^a^	0.227 ± 0.033^b^	0.447 ± 0.123^b^

Khalas	Preflowering	0.426 ± 0.096^a^	0.116 ± 0.006^a^	0.165 ± 0.014^a^	2.158 ± 0.048^a^
	Flowering	0.501 ± 0.092^a^	0.110 ± 0.021^a^	0.263 ± 0.072^a^	1.820 ± 0.039^a^
	Postflowering	0.210 ± 0.063^b^	0.100 ± 0.007^a^	0.049 ± 0.017^b^	1.338 ± 0.068^b^

Lulu	Preflowering	0.526 ± 0.064^a^	1.035 ± 0.066^b^	0.729 ± 0.053^a^	0.935 ± 0.104^a^
	Flowering	0.468 ± 0.045^b^	1.958 ± 0.094^a^	0.341 ± 0.029^b^	0.516 ± 0.046^b^
	Postflowering	0.390 ± 0.017^b^	1.805 ± 0.120^a^	0.658 ± 0.035^a^	0.619 ± 0.172^b^

SOD: superoxide dismutase, CAT: catalase, POX: peroxidase, and APX: ascorbate peroxidase.

Means with different letters in the same variety indicate significantly different values at *p* < 0.05.
